# SARS-CoV-2 incidence monitoring and statistical estimation of the basic and time-varying reproduction number at the early onset of the pandemic in 45 sub-Saharan African countries

**DOI:** 10.1186/s12889-024-18184-8

**Published:** 2024-02-26

**Authors:** Michael Safo Oduro, Seth Arhin-Donkor, Louis Asiedu, Damazo T. Kadengye, Samuel Iddi

**Affiliations:** 1 Pfizer Research & Development, PSSM Data Sciences, Pfizer, Inc, Groton, CT USA; 2 Department of Applied Statistics and Research Methods, University of Northern Colorado, Greeley, Colorado USA; 3 Market Finance Analysis - Sr - Prd - Regional, Humana Inc., Louisville, Kentucky USA; 4 Department of Statistics and Actuarial Sciences, University of Ghana, Accra, Ghana; 5 Data Synergy and Evaluation, African Population and Health Research Center, Manga Close, Nairobi, Kenya; 6 Department of Economics and Statistics, Kabale University, Kabale, Uganda

**Keywords:** COVID-19, Infectious disease, Basic reproduction number, Sub-Saharan Africa, Transmission

## Abstract

The world battled to defeat a novel coronavirus 2019 (SARS-CoV-2 or COVID-19), a respiratory illness that is transmitted from person to person through contacts with droplets from infected persons. Despite efforts to disseminate preventable messages and adoption of mitigation strategies by governments and the World Health Organization (WHO), transmission spread globally. An accurate assessment of the transmissibility of the coronavirus remained a public health priority for many countries across the world to fight this pandemic, especially at the early onset. In this paper, we estimated the transmission potential of COVID-19 across 45 countries in sub-Saharan Africa using three approaches, namely, $R_{0}$ based on (i) an exponential growth model (ii) maximum likelihood (ML) estimation and (iii) a time-varying basic reproduction number at the early onset of the pandemic. Using data from March 14, 2020, to May 10, 2020, sub-Saharan African countries were still grappling with COVID-19 at that point in the pandemic. The region’s basic reproduction number ($R_{0}$) was 1.89 (95% CI: 1.767 to 2.026) using the growth model and 1.513 (95% CI: 1.491 to 1.535) with the maximum likelihood method, indicating that, on average, infected individuals transmitted the virus to less than two secondary persons. Several countries, including Sudan ($R_{0}$: 2.03), Ghana ($R_{0}$: 1.87), and Somalia ($R_{0}$: 1.85), exhibited high transmission rates. These findings highlighted the need for continued vigilance and the implementation of effective control measures to combat the pandemic in the region. It is anticipated that the findings in this study would not only function as a historical record of reproduction numbers during the COVID-19 pandemic in African countries, but can serve as a blueprint for addressing future pandemics of a similar nature.

## Introduction

The 2019 novel coronavirus disease (COVID-19) is a contagious infections disease caused by severe acute respiratory syndrome coronavirus 2 (SARS-CoV-2). The virus can be transmitted through droplets from the nose and mouth when an infected person sneezes, coughs or speaks. A person can contract COVID-19 when they ingest the virus after touching infected surfaces. The first case was identified in Wuhan, the Hubei province in China, at the end of 2019 [1]. It was declared a Public Health Emergency of International Concern (PHEIC) on January 30, 2020, by the World Health Organization (WHO) [2]. Since then, the pandemic spread to about 229 countries and territories around the world with more than 6,960,783 global deaths out of over 771,151,224 confirmed cases as at 4th October, 2023 as inferred from the COVID-19 Data Repository of the World Health Organization [3].

As the COVID-19 pandemic accelerated across the world, Africa was not spared. After recording its first case in Egypt on February 14, 2020, the continent now has a total of about 9,570,365 confirmed coronavirus cases with 175,435 deaths as of 12th October, 2023 according to the COVID-19 Data Repository of the World Health Organization [3]. At the onset of the pandemic, South Africa, Egypt, Morocco, Algeria, Ghana, Nigeria and Cameroon were among countries with the highest number of recorded cases in Africa [1]. Despite the initial importation of most early COVID-19 cases in Africa from Europe, there was a shift towards a majority of recent cases originating within local communities, indicating community-level transmission. While the continent initially experienced a slower rise in cases compared to other regions, it remained susceptible to the pandemic due to a lack of preparedness and limited resources for containment and mitigation efforts. Following the guidelines provided by the World Health Organization (WHO), countries had the potential to curb the virus’s spread by actively monitoring and detecting cases at an early stage. This would be achieved through the implementation of effective strategies designed to isolate and manage cases, as well as to facilitate thorough contact tracing.

Several strategies such as lockdowns and curfews were imposed in countries like South Africa, Ghana, Nigeria, DR Congo among others despite the huge economic and social risks involved. Again, many more countries closed their air and land borders in order to contain the spread of the virus [4]. Airlines across Africa ceased operations to the United States and other nations designated as COVID-19 hotspots. While these actions may not have entirely halted the virus’s transmission within local communities, they represented a valuable step in the effort to contain its spread. [5]. Some governments introduced interventions aimed at limiting person to person contact such as placing a ban on social gatherings including conferences, workshops, funerals, festivals, political rallies, religious activities and other related events in an attempt to stop the onward spread of the virus. Africa’s heavily populated housing and market structures, poor access to safe water and sanitation facilities, and weak health systems, made it difficult to carry out basic measures like social distancing and hand washing effectively. That notwithstanding, it was evident that some countries were ahead of others when it came to implementing containment measures.

At the early stages of a pandemic such as COVID-19, an accurate assessment of the transmissibility of the disease is a top public health priority for many countries. This is critical as it informs governments of the timing and requisite interventions or containment efforts needed. Recent theoretical work has focused on making the best use of data from the initial exponential phase of growth of the incidence in large populations [6, 7]. The reproduction number ($R_0$) is a crucial epidemiological metric in quantifying disease transmission as it represents the number of secondary infections resulting from a primary case in a completely susceptible population [8].The reproduction number ($R_0$) plays a pivotal role during the early stages of a pandemic, offering critical insights for effective public health response.

Firstly, it helps public health officials and researchers gauge the potential of a new infectious disease to cause a pandemic. A high ($R_0$) suggests that the disease is highly contagious and could spread rapidly through the population [9]. Secondly, it guides resource allocation. Understanding $R_0$ helps plan healthcare resources effectively, preventing potential healthcare system overload. Also, $R_0$ shapes intervention strategies. Diseases with higher $R_0$ values require more extensive and aggressive interventions to control their spread [10]. Furthermore, it aids in predicting case trajectories, allowing epidemiologists to estimate the potential course of an outbreak [11]. It also monitors intervention effectiveness - a decreasing $R_0$ indicates success, while an increasing $R_0$ suggests a need for more aggressive measures.

Alimohamadi, Taghdir & Sepandi [12] conducted a study to determine the reproduction number ($R_0$) for COVID-19 in China using a random-effects model. The research obtained studies from international databases, including Google Scholar and Science Direct. The results showed $R_0$ for COVID-19 as approximately 3.32 (2.81-3.82). Based on the results of the study it was concluded that there was a need to reduce the number of contacts within the population to control the epidemic. Liu, Gayle, Wilder-Smith & Rocklöv [13] also undertook a research for the period January 1, 2020 to February 7, 2020 to review $R_0$. Twelve suitable studies from China and other countries which estimated basic reproduction number were obtained from PubMed, bioRxiv and Google Scholar. This review found that the estimated mean $R_0$ for COVID-19 is around 3.28, with a median of 2.79. Further research has been conducted by Zhao et al. [14] to estimate the $R_0$ of novel COVID-19 in China, from 2019 to 2020. It was found that the mean estimate of $R_0$ for the COVID-19 ranges from 2.24 to 3.58 and is significantly larger than 1. This indicated the potential of COVID-19 to cause outbreaks. To examine the growth rate of the outbreak, Shim et al. [15] conducted a study to report the $R_0$ of COVID-19 in South Korea. The daily confirmed cases of COVID-19 in South Korea were extracted from publicly available sources. It was estimated that $R_0$ for COVID-19 was 1.5 with 95% CI (1.4,1.6). The results indicated an early sustained transmission of COVID-19 in South Korea and supported the implementation of social distancing measures to rapidly control the outbreak.

The studies explored so far estimated $R_0$ for COVID-19 for specified periods of time in China and other countries. The objectives of this study are twofold. Firstly, we focus on estimating the basic reproduction number of 45 sub-Saharan Africa(SSA) countries at the early onset of the pandemic. We define “early onset" in the context of our study as the time between March 14, 2020 and May 10, 2020. Secondly, although a basic initial estimate of the reproduction number is useful, continuous surveillance of this parameter over time provides useful feedback to governments or agencies on the efficacy of interventions and containment efforts or the need to tighten control efforts to bring the pandemic under control. In this vein, time-varying reproduction numbers $R_{t}$ over tri-weekly periods for SSA as a whole and some specific SSA countries at the early onset of the COVID-19 pandemic are also estimated. As of April, 2020, governments of some sub-Saharan African countries, for example, Ghana, had already relaxed locked down containment efforts. Did African countries win the war against COVID-19 at the early onset? Basic reproduction and time-varying reproduction numbers can serve as a useful epidemiological metric in measuring the spread of the virus in Africa. This can aid governments in formulating and making decisions on the extent to which public health interventions should be relaxed or strengthened in the future for similar pandemics. We anticipate that the results in this study serve not only as an archived record of reproduction numbers for the COVID-19 pandemic in African countries, but serve as a guide for similar future pandemics.

## Data and methods

### Data source

The data supporting the findings of this study were sourced from archived data generated by the COVID-19 Data Repository by the Center for Systems Science and Engineering (CSSE) at the Johns Hopkins University [16]. The data used accross 45 African countries included confirmed and active COVID-19 cases from March 14, 2020 to May 10, 2020.

### Estimation process of the exponential growth rate

In calculating the basic reproduction number, $R_{0}$, an estimate of the epidemic growth rate is first computed. It is widely known that for the initial phase of respiratory infectious disease outbreaks, the recorded incidence follows an exponential trajectory [17]. The rate of this exponential growth reflects some sort of severity metric of the outbreak, and can be described as the “per capita" change in the number of new cases per unit time. The growth rate parameter, when computed, can be related to the basic reproduction number through a moment generating function. Here, we estimate the growth rate from a log-linear model via a transformation of an exponential relation. If the number of cases in time is expected to follow an exponential relation, then

1 $$\begin{aligned} y = \alpha _{0}\exp (\alpha _{1} t) \end{aligned}$$

which can be log transformed as

2 $$\begin{aligned} \text {log}(y) = \text {log}(\alpha _{0}) + \alpha _{1}t \end{aligned}$$

where, *y*, represents the recorded incidence, $\alpha _{1}$ is the growth rate, *t* is the number of days since a specific point in time, which is usually recorded from when the outbreak started, and $\text {log}(\alpha _{0})$ is the intercept of the log-linear model. Using the ordinary least squares estimation approach, the growth rate can be estimated. For clarity in derivation, we represent the intercept, $\text {log}(\alpha _{0})$, as $\phi$. The least squares problem is that of finding the growth parameter, $\alpha _{1}$ and then $\phi$, such that the function

3 $$\begin{aligned} G\left( \phi , \alpha _{1}\right) = \sum \limits _{i=1}^{n}\left( \alpha _{1} t_{i}+ \phi -\text {log}y_{i}\right) ^{2} \end{aligned}$$

is minimized. The minimum of the quadratic objective function in (3), which is $\hat{\alpha }_{1}$ and $\hat{\phi }$ can be achieved by obtaining the partial derivatives of $\alpha _{1}$ and $\phi$ below;

4 $$\begin{aligned} \frac{\partial G}{\partial \phi }=\sum \limits _{i=1}^{n} 2\left( \alpha _{1} t_{i}+\phi -\text {log}y_{i}\right) ,\quad \frac{\partial G}{\partial \alpha _{1}}=\sum \limits _{i=1}^{n} 2t_{i}\left( \alpha _{1} t_{i}+\phi -\text {log}y_{i}\right) \end{aligned}$$

The partial derivatives, when set to zero yield a system of linear equations

5 $$\begin{aligned} \begin{aligned} n\phi +\left( \sum \limits _{i=1}^{n} t_{i}\right) \alpha _{1}&=\sum \limits _{i=1}^{n} \text {log}y_{i} \\ \left( \sum \limits _{i=1}^{n} t_{i}\right) \phi +\left( \sum \limits _{i=1}^{m} t_{i}^{2}\right) \alpha _{1}&=\sum \limits _{i=1}^{n} t_{i}\text {log}y_{i} \end{aligned} \end{aligned}$$

Solving the system of equations yields, first, the intercept as

6 $$\begin{aligned} \hat{\phi }=\dfrac{\left( \sum \limits _{i=1}^{n} t_{i}^{2}\right) \left( \sum \limits _{i=1}^{n} \text {log}y_{i}\right) -\left( \sum \limits _{i=1}^{n} t_{i}\right) \left( \sum \limits _{i=1}^{n} t_{i} \text {log}y_{i}\right) }{n \sum \limits _{i=1}^{n} t_{i}^{2}-\left( \sum \limits _{i=1}^{n} t_{i}\right) ^{2}} \end{aligned}$$

And then the estimated exponential growth rate, $\alpha _{1}$,as

7 $$\begin{aligned} \hat{\alpha }_{1}=\dfrac{n \sum \limits _{i=1}^{n} t_{i} \text {log}y_{i}-\left( \sum \limits _{i=1}^{n} t_{i}\right) \left( \sum \limits _{i=1}^{n}\text {log}y_{i}\right) }{n \sum \limits _{i=1}^{n} t_{i}^{2}-\left( \sum \limits _{i=1}^{n} t_{i}\right) ^{2}} \end{aligned}$$

### Estimation process of the basic reproduction number

An important variable in infectious disease modeling is the basic reproduction number, $R_{0}$, which represents the number of secondary infections resulting from a primary case in a completely susceptible population. The value of the reproductive number can be indirectly estimated from the exponential growth rate of the disease [18]. A relationship between the reproductive number and the growth rate is thus established via a moment generating function of a so-called generation time distribution, also called a serial interval. The serial time distribution characterizes the distribution of the time lag between infection in a primary case and a secondary case. More precisely, the relationship between the exponential growth rate $\hat{\alpha }_{1}$ and reproductive number $R_{0}$ is premised on the shape of the serial interval distribution. In this study, the relationship between $\hat{\alpha }_{1}$ and $R_{0}$ is given as

8 $$\begin{aligned} R_{0} = \dfrac{1}{M(-\hat{\alpha }_{1})} \end{aligned}$$

where *M* represents the moment generating function of the serial time distribution which is discrete. In this study, this serial interval distribution is assumed to be a Gamma distribution. Based on this information we derive the $R_{0}$ mathematically, by first deriving an expression for $M(\hat{\alpha }_{1})$. The moment generating function (MGF) of the Gamma distribution is obtained as a function of $-\hat{\alpha }_{1}$. Generally, the moment generating function is defined as

9 $$\begin{aligned} M_{Y}(t)=\int _{-\infty }^{\infty } \exp ({ty}) f_{Y}(y) \textrm{d} y \end{aligned}$$

where $f_Y(y)$ is the probability density function of the random variable *Y*.The moment generating function of the Gamma distribution, with $\alpha$ and $\beta$ as shape and scale parameters respectively is defined as;

10 $$\begin{aligned} \frac{\beta ^{\alpha }}{\Gamma (\alpha )} \int _{0}^{\infty } y^{\alpha -1} \exp \left( -(\beta -t) y \right) \textrm{d}y = \displaystyle \left( \dfrac{\beta -t}{\beta }\right) ^{-\alpha } \end{aligned}$$

As a function of $-\hat{\alpha }_{1}$, the MGF is expressed as

11 $$\begin{aligned} M(-\hat{\alpha _{1}})=\left( \dfrac{\beta +\hat{\alpha }_{1}}{\beta }\right) ^{-\alpha } \end{aligned}$$

Hence, the basic reproduction number can be estimated as

12 $$\begin{aligned} R_{0} = {\left( 1+\dfrac{\hat{\alpha }_{1}}{\beta } \right) ^{\alpha }} \end{aligned}$$

Notably, the parameters $\alpha$ and $\beta$ are strictly positive (that is $\alpha$ and $\beta$ should be greater than zero). Another constraint on the relationship is that $\hat{\alpha }_{1} > \beta$.

As a form of a sensitivity analysis, the basic reproduction number is also computed using a maximum likelihood approach. This approach, first proposed by White & Pagano[19] assumes that the number of secondary cases caused by a primary case is Poisson distributed with expected value $R_{0}$. Given daily incidence recorded over time, $\left( I_{0},I_{1},I_{2}, \cdots ,I_{T}\right)$ and a serial interval $s$, $R_{0}$ can be estimated by maximizing the likelihood

$$\begin{aligned} L\left( R_{0}, s\right) =\prod _{t=1}^{T} \frac{\textrm{exp}{(-\mu _{t})} \mu _{t}^{I_{t}}}{I_{t} !} \end{aligned}$$

where $$\mu _{t}=R_{0}\sum \limits _{i=1}^{t} I_{t-i} s_{i}$$.

### Estimation process of the time-varying reproduction number

The reproduction number obtained in the previous section is constant in time and context specific. When an infectious disease is spreading through a population, it is often more plausible to work with a time-varying reproduction number also known as the effective reproductive number, $R_t$. This statistic describes the average number of secondary infections that can arise from a primary case on a day-by-day basis. This metric can be a useful indicator in quantifying the transmissibility of the disease and the assessment of the effectiveness of public health interventions. For example, during an epidemic, if a country’s reproduction number declines over a significant amount of time, it would indicate that probably the governments control efforts are efficient. Typically, governments would want the $R_{0}$ values to be less than 1 over time. In this article, we compute time-varying reproduction numbers over tri-weekly windows. We follow the time varying reproduction estimation approach by Cori et al. [20] and White & Pagano [19].

In the initial phase of an epidemic, collected surveillance and contact tracing data are counts and constitute incidence data. Due to the count nature of the incidence, we can assume that the rate at which persons are infected and their infectiousness profile through time is a Poisson process. After a person is infected, their given infectious profile through time is characterized by a probability distribution $\theta _{p}$, which depends on the time, *p* since an infection of a person. However, this is independent of the calendar time, *t*. The rate at which infected person at a time, $t-p$ gives rise to new infections in time step *t* is $R_{t}\theta _{p}$, where $R_{t}$ is the time varying infection reproduction number. The time varying reproduction number can be estimated as

13 $$\begin{aligned} R_{t}=\frac{\lambda _{t}}{\sum \limits _{p=1}^{t}\lambda _{t-p}\theta _{p}} \end{aligned}$$

which is the ratio of newly generated incidences, $\lambda$, at time *t* to the sum of incidences (sum of infections) up to time step $t-1$, weighted by the probability distribution of the infectiousness profile, $\theta _{p}$. It is worthy of note that $\theta _{p}$ is a serial distribution that sums up to one. The expected value (average) of incident cases of at time *t* is also given as

14 $$\begin{aligned} E(\lambda _{t})=R_{t}{\sum \limits _{p=1}^{t}\lambda _{t-p}\theta _{p}} \end{aligned}$$

Let $\mu$ represent $R_{t}{\sum \limits _{p=1}^{t}\lambda _{t-p}\theta _{p}}$ for clarity in derivation. The likelihood of an incidence at time *t* given the reproduction number conditioned on previous incidences, can be defined, based on a Poisson likelihood as

15 $$\begin{aligned} P\left( \lambda _{t} |\lambda _{0},\lambda _{1},\lambda _{2} \ldots , \lambda _{t-1}, R_{t},\theta \right) =\frac{\left( R_{t} \mu _{t}\right) ^{\lambda _{t}} \exp {(-R_{t} \mu _{t})}}{\lambda _{t} !} \end{aligned}$$

Since there could be high variability in the $R_{t}$ estimates, computations of this likelihood can be done over longer time windows, as it has been established that there is difficulty in interpretation for smaller time windows [21]. If we compute $R_{t}$ estimates assuming that it is constant within a time window, $\nu$, we can obtain estimates of the time-varying reproduction number, $R_{t,\nu }$ at each time step, *t* over a time window $\nu$ which ends at time, *t*. For the time-varying reproduction number, $R_{t,\nu }$, the likelihood of the incidence, $\lambda _{t-\nu +1},\dots ,\lambda _{t}$ in this time frame given $R_{t,\nu }$ and conditioned on previous observed incidences can be defined as

16 $$\begin{aligned} P\left( \lambda _{t-\nu +1}, \ldots , \lambda _{t} | \lambda _{0},\lambda _{1},\lambda _{2},\lambda _{3} \ldots , \lambda _{t-\nu }, \theta , R_{t, \nu }\right) =\prod _{p=t-\nu +1}^{t} \frac{\left( R_{t,\nu } \mu _{p}\right) ^{\lambda _{p}} \exp (-R_{t,\nu } \mu _{p})}{\lambda _{p}!} \end{aligned}$$

Using Bayesian Inference, we can obtain average time-varying reproduction numbers, $\hat{R}_{t,\nu }$ with their corresponding variances and credible intervals for each time window via a joint posterior distribution of $\hat{R}_{t,\nu }$ and under the assumption of a Gamma prior distribution with scale and shape parameters, $\alpha$ and $\beta$. In fact, the assumed prior gamma distribution reflects the serial distribution discussed in the previous section.

The Gamma $\left( \alpha , \beta \right)$ prior distribution for ${R}_{t,\nu }$ is given by

17 $$\begin{aligned} P({R}_{t,\nu })=\frac{1}{\Gamma (\alpha ) \beta ^{\alpha }} {R}_{t,\nu }^{\alpha -1} \exp \left( {-\frac{{R}_{t,\nu }}{\beta }}\right) ,~~~~0< R_{t,v} < \infty \end{aligned}$$

with the likelihood of the incidence, $\lambda _{t-\nu +1},\dots ,\lambda _{t}$ in the time frame given $R_{t,\nu }$ conditioned on previous observed incidences already specified in (16). The joint posterior density of ${R}_{t,\nu }$ is given as

18 $$\begin{aligned} P\left( \lambda _{t-\nu +1}, \ldots , \lambda _{t}, R_{t, \nu }| \lambda _{0}, \ldots , \lambda _{t-\nu }, \theta \right) = \prod _{p=t-\nu +1}^{t} \frac{\left( R_{t,\nu } \mu _{p}\right) ^{\lambda _{p}} \exp (-R_{t,\nu } \mu _{p})}{\lambda _{p}!}\frac{{R}_{t,\nu }^{\alpha -1} \exp \left( {-\frac{{R}_{t,\nu }}{\beta }}\right) }{\Gamma (\alpha ) \beta ^{\alpha }} \end{aligned}$$

19 $$\begin{aligned} = R_{t, \nu }^{\nu (\alpha -1)+\sum \limits _{p=t-\nu +1}^{t}\lambda _{p}}\exp \left[ {-R_{t,\nu }\left( \sum \limits _{p=t-\nu +1}^{t}\mu _{p}+\frac{1}{\beta }\right) }\right] \prod _{p=t-\nu +1}^{t} \frac{\mu _{p}^{\lambda _{p}}}{\lambda _{p} !} \frac{1}{\Gamma (\alpha )\beta ^{\alpha }} \end{aligned}$$

20 $$\begin{aligned} \propto R_{t, \nu }^{\nu (\alpha -1)+\sum \limits _{p=t-\nu +1}^{t}\lambda _{p}}\exp \left[ {-R_{t,\nu }\left( \sum \limits _{p=t-\nu +1}^{t}\mu _{p}+\frac{1}{\beta }\right) }\right] \prod _{p=t-\nu +1}^{t} \frac{\mu _{p}^{\lambda _{p}}}{\lambda _{p} !} \end{aligned}$$

Expression (20) is the kernel of a ${\text {Gamma}}$ distribution,

21 $$\begin{aligned} {\text {Gamma}}\left( \nu \alpha -\nu +1+\sum \limits _{p=t-\nu +1}^{t}\lambda _{p}, \dfrac{1}{\sum \limits _{p=t-\nu +1}^{t}\mu _{p}+\frac{1}{\beta }}\right) \end{aligned}$$

Hence, the posterior mean and variance of this distribution of $R_{t,\nu }$ can be respectively given as

22 $$\begin{aligned} \text {Posterior Mean of }R_{t,\nu }=\dfrac{\nu \alpha -\nu +1+\sum \limits _{p=t-\nu +1}^{t}\lambda _{p}}{\sum \limits _{p=t-\nu +1}^{t}\mu _{p}+\frac{1}{\beta }} \end{aligned}$$

23 $$\begin{aligned} \text {Posterior variance of }R_{t,\nu }=\dfrac{\nu \alpha -\nu +1+\sum \limits _{p=t-\nu +1}^{t}\lambda _{p}}{\left( \sum _{p=t-\nu +1}^{t}\mu _{p}+\frac{1}{\beta }\right) ^{2}} \end{aligned}$$

The estimation is carried using the R function estimate_R() in the EpiEstim package [20]. To encourage reproducibility and open science, the code used for producing the figures in this article can be found in the GitHub Repository: https://github.com/IddiSam/Covid19SSA.git.

## Results and discussions

**Fig. 1:**
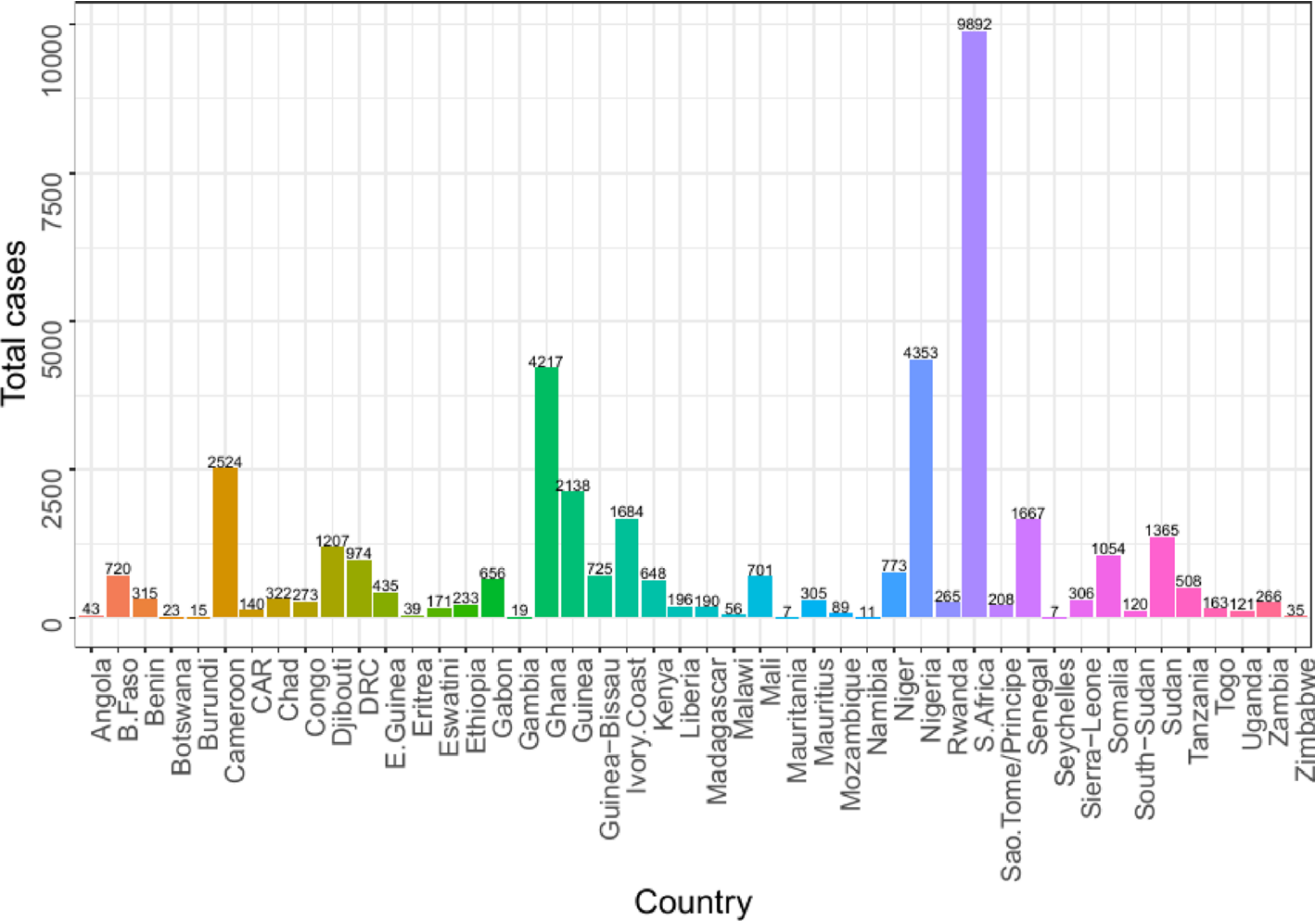
Total number of confirmed COVID-19 cases in countries across sub-Saharan Africa as at May 10, 2020

**Fig. 2:**
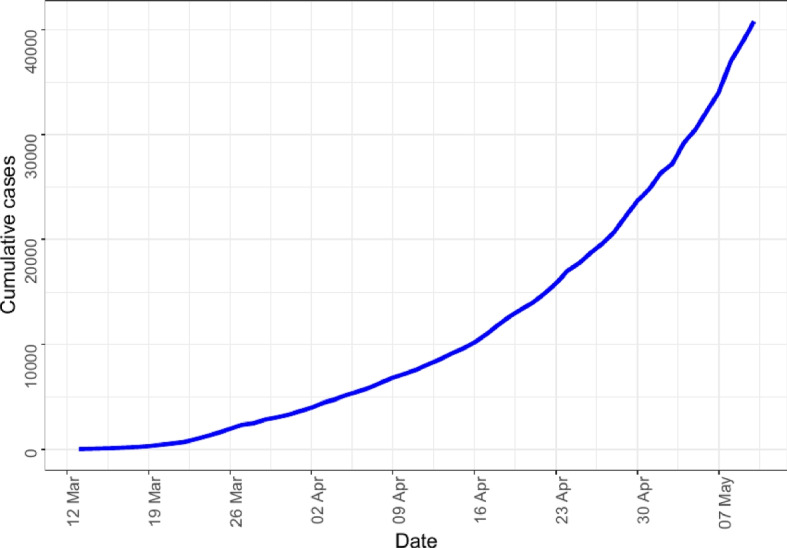
Cumulative number confirmed COVID-19 cases in Sub-Saharan Africa from March 14, 2020 to May 10, 2020

**Fig. 3:**
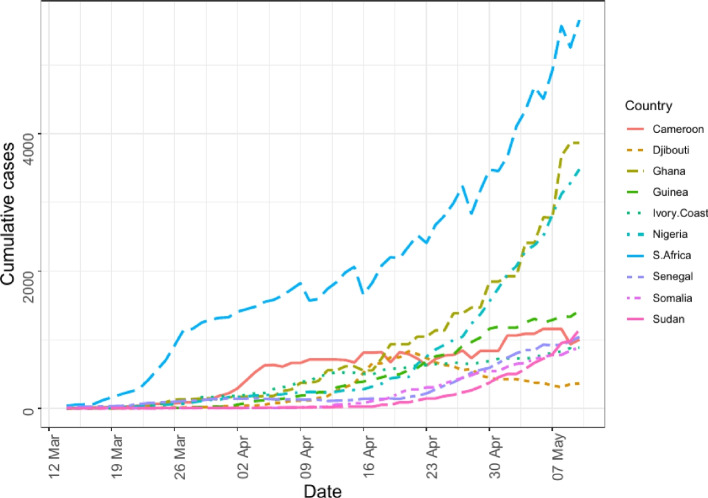
Top 10 countries with the highest cumulative number of confirmed COVID-19 cases in sub-Saharan Africa as at May 10, 2020

To gain insight into the COVID-19 data retrieved from the COVID-19 Data Repository by the Center for Systems Science and Engineering (CSSE) at Johns Hopkins University, an exploratory data analysis was performed. Graphical techniques were used. As can be observed from Fig. 1, as of May 10, 2020, top 10 African countries with the highest cumulative number of COVID-19 cases were South Africa (9892), Nigeria (4352), Ghana (4217), Cameroon (2524), Guinea (2138), Ivory Coast (1884), Senegal (1667), Sudan (1365), Djibouti (1207), and Somalia (1054) in order of magnitude. From Fig. 2 it is observed that between March 14, 2020 and May 10, 2020 the cumulative number of COVID-19 cases has monotonically increased in sub-Saharan Africa. A trend of the cumulative number of confirmed COVID-19 cases for these 10 countries from March 14, 2020, to May 10, 2020, can be seen in Fig. 3. It is observed in Fig. 3 that South Africa consistently recorded the highest cumulative number of COVID-19 cases throughout the period. Djibouti had seen a steady decline in COVID-19 cases from April 22 to May 7. There was a fairly slow increase in COVID-19 cases for Ghana till cases shot up on April 9; the same could be said of Nigeria, Ivory Coast and Guinea. Cases in Sudan seem to have been stable since April 4. In general, it did not seem like the continent had reached its peak of the pandemic yet. The epidemic plots observed so far show evidence of a steady increase in incidence reflecting an exponential trend, as would be expected for a pandemic spread. If plotted on a logarithmic axis, one would see a linear increase on a log scale if the epidemic curve is accurately exponential. A log-linear model is thus fitted to the total SSA and country-specific incidence data. From the model results, we extract the growth rate prior *r* to the peak (based on the current data) as 0.098 per day with a $95\%$ CI of (0.0869, 0.1104). The decay rate can be computed but not informative as SSA is still in the early phase of the outbreak. The growth rate had a corresponding doubling time of 7.0223 days with $95\%$ CI (6.2769, 7.9687) days.

**Fig. 4:**
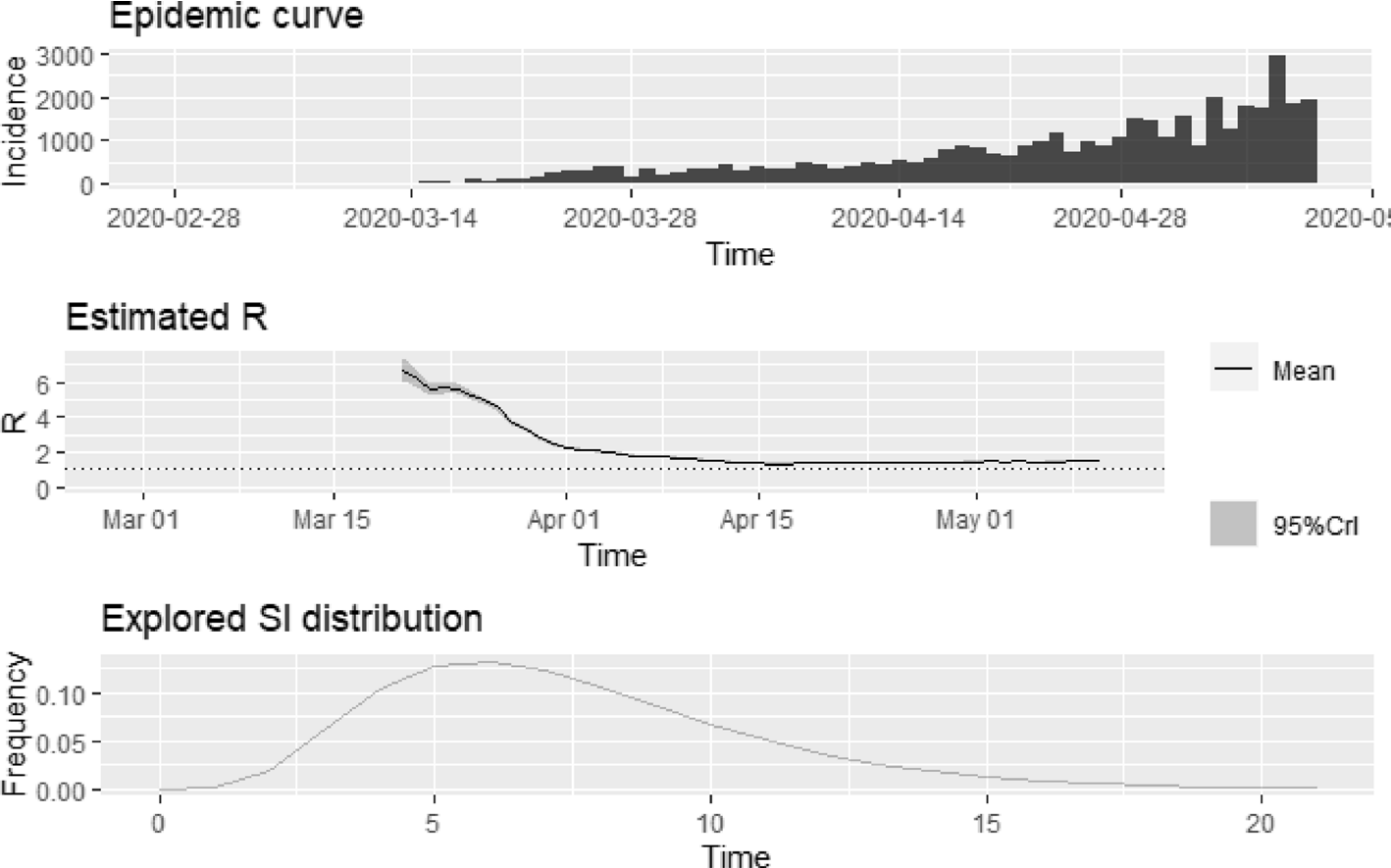
Plot of the time-varying reproduction number of COVID-19 cases in Sub-Saharan Africa from start to May 10, 2020

**Fig. 5:**
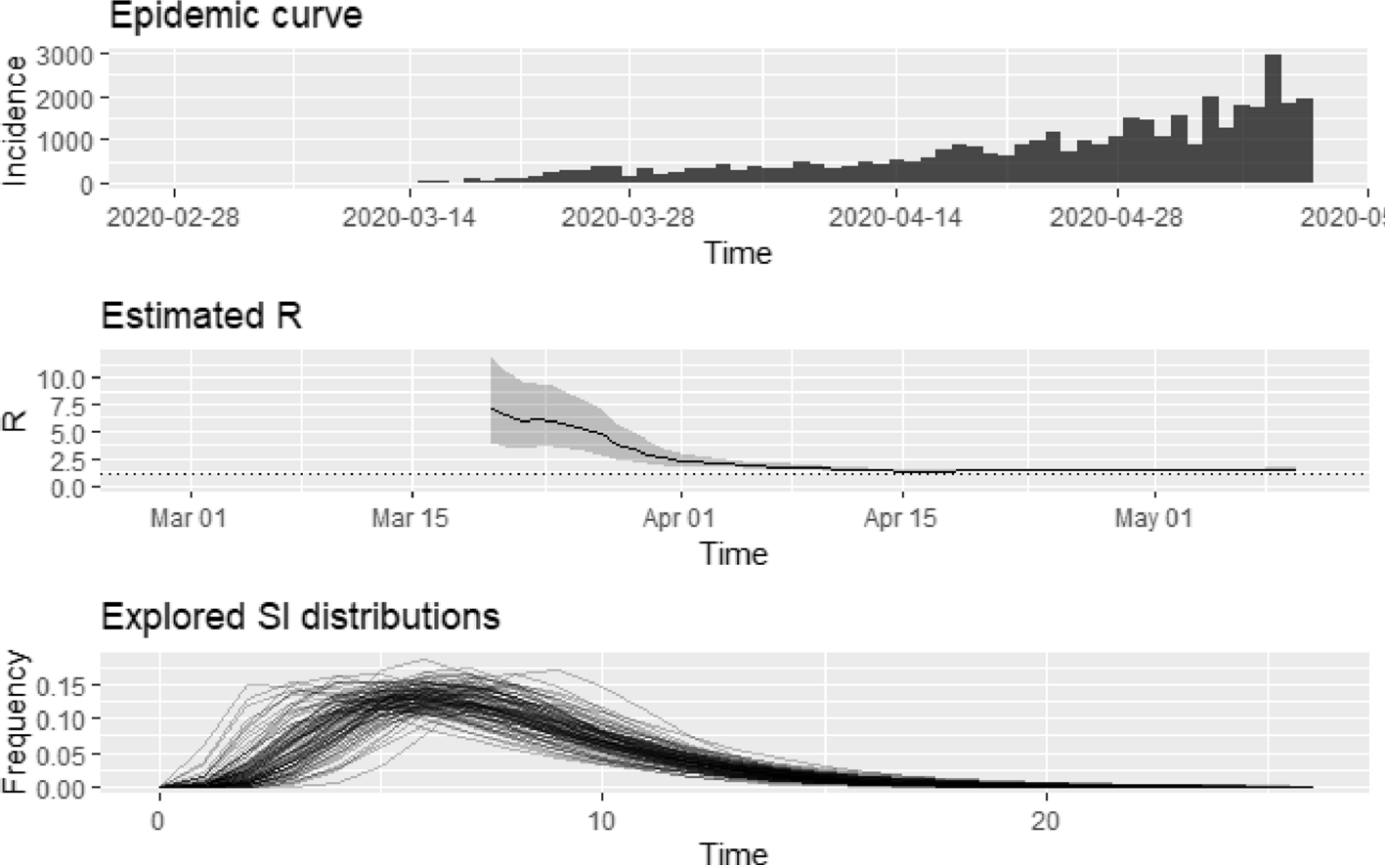
Plot of the time-varying reproduction number of COVID-19 cases in SSA as at May 10, 2020 accounting for uncertainty in the serial interval distribution

An overall basic reproduction number is obtained for the 45 SSA countries, via the relationship between the growth rate obtained and the moment generating function of the serial interval distribution. The mean serial interval used in this article is 7.5 days with a standard deviation of 3.4 days. This mimics that obtained by Li et al. [22] who estimated the serial interval of the COVID-19 pandemic as 7.5 days with 95% CI, (5.3, 19), in its early stages at Wuhan, China. Based on the epidemic growth rate, $\hat{\alpha }_{1}$, the overall basic reproduction number estimated for the SSA countries as of 10th May, 2020, was 1.89, with 95% CI, (1.767, 2.026). As a form of sensitivity, this basic reproduction number estimation is once again done via maximum likelihood. This yields a reproduction number for SSA as 1.513 with 95% CI, (1.491, 1.535). This basic reproduction number is quite conservative but still in line with that computed based on the growth rate. In order to track and assess the effectiveness of intervention and containment efforts by African governments, the time-varying reproductive number is estimated. We employ the “EpiEstim" package in R software for this modeling process [20]. The serial interval distribution used here is simulated using a discrete gamma distribution with a mean 7.5 days and standard deviation 3.4 days. The daily estimates of the time-varying reproduction numbers $R_{t}$ over a 3-week sliding window from outbreak start in Africa are plotted in Fig. 4 with the grey area depicting the $95\%$ credible intervals. The horizontal dashed line reflect the threshold R-value of 1. Inferring from the plots in Fig. 4, an observed decline in the slope of the estimated $R_{t}$ curve is observed by the end of March, probably indicative of the influence of containment efforts by sub-Saharan Africa governments in reducing transmission of the disease. However, by April 15th, 2020 an increase in the time-varying reproduction number is observed. This could be attributed to more testing or contact tracing efforts by governments. It is however worthy of note that COVID-19 is transmissible before onset of symptoms and hence there could be the likelihood that non-symptomatic spreaders of the disease may have gone undetected from the onset of the disease. Between the week of 20th April and 10th May, the average instantaneous reproduction number was 1.46 with $95\%$ CI (1.44, 1.48), indicating that infected persons were infecting less than 2 secondary persons in sub-Saharan Africa. The time-varying reproduction number is once again computed, but this time the uncertainty of the serial interval distribution specified is accounted for. In doing this, the mean and the standard deviation of the serial interval are each drawn from a truncated normal distribution, with parameters specified. Here, we specify the mean of the serial interval distribution as a Normal (7.5, 10), truncated at 1 and 10 and the standard deviation of the serial interval as a Normal (3.4, 5.2), truncated at 0.5 and 5.2. The result is shown in Fig. 5 below. The simulated result above look reasonable, and clearly in line with the result first shown in Fig. 4. The patterns observed indicate that no matter the distribution used for the serial interval, similar instantaneous intervals are observed. The results so far obtained are from pooled incidence data from all 45 countries considered. We obtain the basic reproduction number estimates based on the epidemic growth rate for each selected country.

**Fig. 6:**
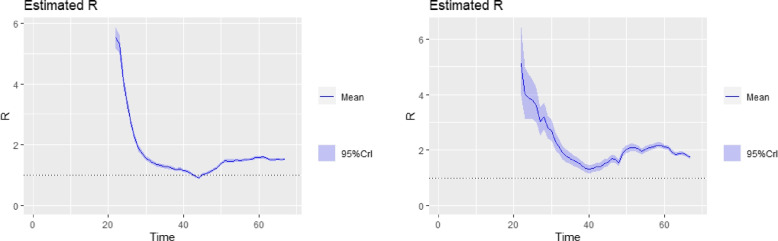
Time Varying Reproductive Number Plots for tri-weekly sliding window. **left:** South Africa, **right:** Nigeria

**Fig. 7:**
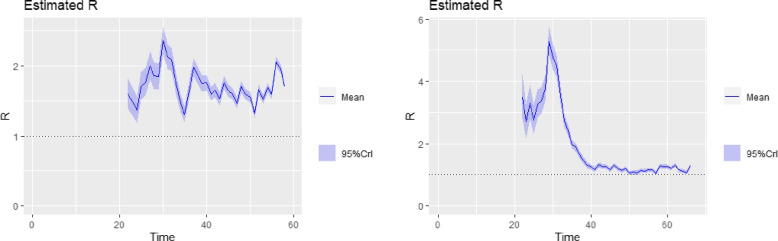
Time Varying Reproductive Number Plots for tri-weekly sliding window. **left:** Ghana, **right:** Cameroon

**Fig. 8:**
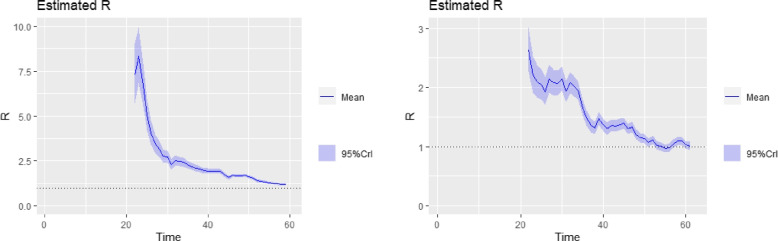
Time Varying Reproductive Number Plots for tri-weekly sliding window. **left:** Guinea, **right:** Ivory Coast

The tabulated results are shown in Table 1. Inference from Table 1 (for $R_{0}$ values based on log-linear model ) showed that the country with the highest basic reproduction number as of 10th May, 2020 was Sudan with an $R_{0}$ of $2.03~(95\%~ \text {CI}~1.83, 2.25)$. This is closely followed by Ghana $1.87 ~(95\% ~\text {CI}~1.71, 2.08)$, and Somalia, $1.85~(95\% \text {CI}~1.69, 2.04)$. South Africa, Nigeria, Chad, Benin and a host of other SSA countries have an $R_{0}$ greater than 1. In terms of countries with lower $R_{0}$ on average, Mauritius, Burkina Faso, Eritrea and Madagascar lead the chart with $R_{0}$ values of 0.7, 0.96, 0.94, 0.97 respectively. This implied that COVID-19 seem to be spreading at a relatively lower rate in these countries compared to the other countries. On average, infected persons in these countries were in turn infecting less than one person. The countries with higher $R_{0}$ had to continue to tighten their containment efforts rather than lift restrictions. Plots of the time- varying reproduction number for tri-weekly windows are obtained for the top 6 countries with very high confirmed cases and shown in Figs. 6, 7 and 8. The time-varying reproduction number plots based on the 3-week sliding window for the top six countries reveal an interesting trend. Over time, a steady decline in the $R_{t}$ estimates for the countries is observed. By day 40 after the disease outbreak, $R_{t}$ trajectory for Cameroon, South Africa, Guinea and Ivory-Coast seem to stabilize. In contrast, there was a steady increase in the trajectory for Ghana and Nigeria as evidenced in their plots. Over the last three weeks prior to May 10th, the reproduction number seem to hover above the R threshold of 1 in all top six countries suggesting that community infections in these selected countries were still causing continuous spread albeit government intervention measures.

**Table 1:** Basic Reproduction Number Estimates obtained for 45 Subsaharan African Countries based on a Log-Linear Model to recorded data up until 10th May, 2020

Country	Growth Rate	CI for GrowthRate	${\boldsymbol R}_{\mathbf0}$	CI for ${\boldsymbol R}_{\mathbf0}$
Angola	0.017	(0,0.0335)	1.12	(0.95,1.23)
Burkina Faso	-0.005	(-0.0196,0.0097)	0.96	(0.88,1.06)
Benin	0.066	(0.051,0.0814)	1.57	(1.38,1.69)
Botswana	-0.007	(-0.0719,0.0575)	0.96	(0.62,1.4)
Burundi	0.041	(-0.0148,0.0971)	1.3	(0.76,1.88)
Cameroon	0.053	(0.0293,0.0757)	1.44	(1.26,1.64)
CAR	0.052	(0.0158,0.0877)	1.43	(1.2,1.77)
Chad	0.080	(0.0573,0.1027)	1.72	(1.46,1.89)
Congo	0.038	(0.0094,0.0659)	1.28	(1.06,1.56)
Djibouti	0.024	(-0.0048,0.0529)	1.18	(0.94,1.41)
DRC	0.050	(0.0365,0.0636)	1.41	(1.28,1.52)
Equitorial Guinea	0.076	(0.037,0.1146)	1.65	(1.29,2.06)
Eritrea	-0.006	(-0.0664,0.0536)	0.94	(0.67,1.4)
Eswatini	0.033	(0.014,0.0514)	1.26	(1.13,1.43)
Ethiopia	0.019	(0.0034,0.0342)	1.13	(1.03,1.24)
Gabon	0.076	(0.0553,0.0974)	1.69	(1.48,1.88)
Gambia	0.009	(-0.0147,0.0329)	1.07	(0.88,1.21)
Ghana	0.097	(0.0769,0.1164)	1.87	(1.71,2.08)
Guinea	0.073	(0.0587,0.0867)	1.61	(1.46,1.77)
Guinea-Bissau	0.083	(0.0465,0.1187)	1.76	(1.47,2.1)
Ivory Coast	0.046	(0.0307,0.0604)	1.37	(1.24,1.5)
Kenya	0.037	(0.0265,0.0483)	1.28	(1.21,1.37)
Liberia	0.037	(0.0161,0.0574)	1.27	(1.06,1.49)
Madagascar	-0.005	(-0.0277,0.0175)	0.97	(0.85,1.1)
Malawi	0.012	(-0.0223,0.0454)	1.07	(0.85,1.32)
Mali	0.049	(0.0339,0.0633)	1.38	(1.26,1.5)
Mauritius	-0.048	(-0.0801, -0.0157)	0.7	(0.551,0.878)
Mauritania	-0.002	(-0.0213,0.0181)	0.99	(0.85,1.13)
Mozambique	0.011	(-0.0108,0.0333)	1.09	(0.89,1.27)
Namibia	0.027	(-0.0411,0.0942)	1.19	(0.8,1.7)
Niger	0.002	(-0.0206,0.0249)	1.01	(0.88,1.17)
Nigeria	0.086	(0.0762,0.0955)	1.75	(1.66,1.86)
Rwanda	0.009	(-0.0052,0.024)	1.07	(0.98,1.19)
South Africa	0.066	(0.056,0.0767)	1.55	(1.46,1.66)
Sao Tome and Principe	0.057	(-0.0604,0.1749)	1.57	(0.7,4.18)
Senegal	0.054	(0.0445,0.0639)	1.44	(1.34,1.52)
Sierra-Leone	0.085	(0.0673,0.1027)	1.74	(1.58,1.92)
Somalia	0.094	(0.0749,0.1128)	1.85	(1.69,2.04)
South-Sudan	0.078	(0.0204,0.1359)	1.61	(1.13,2.27)
Sudan	0.110	(0.0931,0.1276)	2.03	(1.83,2.25)
Tanzania	0.086	(0.0527,0.1184)	1.79	(1.46,2.14)
Togo	0.012	(-0.0044,0.0275)	1.08	(0.97,1.2)
Uganda	-0.007	(-0.028,0.014)	0.949	(0.818,1.049)
Zambia	0.028	(0.01,0.0458)	1.21	(1.06,1.34)
Zimbabwe	0.003	(-0.018 0.0238)	1.02	(0.846, 1.149)

It is important to underscore that the estimated basic and time-varying reproduction numbers in this study align closely with findings from other research. Notably, early pandemic research studies in Chad ($R_{0}=1.63$), Central Afican African Republic ($R_{0}=1.40$), Congo($R_{0}=1.41$), Tanzania($R_{0}=1.16$), Angola($R_{0}=1.55$), Malawi($R_{0}=1.55$) and Mozambique($R_{0}=1.25$) by Han et al. [23], employing similar methodologies, yielded estimates that were consistent or in close agreement with our basic reproduction estimates (see Table  1). In comparison to our study, very close estimates of the basic reproduction were also observed in Ivory Coast ($R_{0}=1.47$), Ghana ($R_{0}=1.85$), Kenya, ($R_{0}=1.57$), and Nigeria ($R_{0}=1.91$) from a study conducted by Oshinubi et al. [24] and Demongeot et al. [25].

Additionally, parallel investigations into time-varying reproduction numbers in Ghana [26], Cameroon [27], and Nigeria [28] further supported the outcomes of this study. More specifically, these studies collectively illustrate that from late March to April 2020, the effective/time-varying reproduction numbers predominantly fluctuated between 2.5 and 4 in the early stages of the epidemic, gradually declining to 1 by late April and early May, 2020. It is worth noting that the specific sliding windows employed for these estimates varied across studies.

In order to enhance the practical policy implications of our study, we recognize the importance of delving into the correlation between specific government interventions and the observed changes in both the basic and the time varying reproduction number. Understanding how individual interventions impact the transmission dynamics of the disease can inform evidence-based decision-making and guide the design of targeted public health measures. For instance, in Ghana, as illustrated in Fig. 7 (left), diverse associations between government policies and interventions were observed, influencing the fluctuations in COVID-19 transmission. Specifically, the implementation of social gathering restrictions and travel bans on March 15, 2020 [26], showed negligible changes in virus spread during the 20th to 40th day of the pandemic, despite high levels of time-varying reproduction. However, by April to early May 2020, when the Ghanaian government had mandated border closures by March 22, 2020 [26], reproduction numbers started decreasing, trending towards 1.

Similarly, in Nigeria, travel restrictions were initiated on March 18, 2020 [29], three weeks after the identification of the first index case. This delay potentially allowed for the importation of the virus, especially notable as a majority of confirmed cases were individuals returning from abroad. As seen in Fig. 6(right), this delayed response may have contributed to higher estimated time-varying reproduction numbers initially, but a sharp decline was observed over subsequent three-week sliding windows.

Overall, the sub-Saharan African region experienced relatively higher levels of COVID-19 incidence between early March and April, possibly due to delayed government interventions or containment efforts, as reflected in the time-varying reproduction plot in Fig. 6. Nevertheless, these containment efforts, including travel bans and restrictions, eventually paid off, leading to reproduction numbers leveling off to almost 1 or less.

## Conclusions

In this study, we utilized COVID-19 incidence data sourced from the John Hopkins COVID-19 data repository, covering the period from March 9, 2020, to May 10, 2020. Our primary objective was to estimate the basic reproduction number ($R_0$) for sub-Saharan Africa, employing three distinct analytical approaches: the exponential growth model, the maximum likelihood approach, and the time-varying basic reproduction number estimation approach. This multifaceted methodology enabled us to provide regional and country-specific estimates. Our research findings revealed crucial insights regarding the epidemiological dynamics of COVID-19 in sub-Saharan Africa during the early onset of the pandemic. The calculated basic reproduction number suggested that infected individuals, on average, were transmitting the virus to less than two secondary persons within the specified time frame. Notably, some sub-Saharan countries, such as Sudan, Ghana, and Somalia, exhibited relatively high transmission rates, with $R_0$ values hovering around 2. Similarly, nations including South Africa, Nigeria, Chad, Benin, and numerous others in the region displayed $R_0$ values exceeding 1. These findings implied that without effective intervention measures, the number of COVID-19 cases was likely to increase in these regions.

However, our study also identified sub-Saharan countries, including Mauritius, Burkina Faso, Eritrea, and Madagascar, as leading the way in terms of containing the virus. These countries exhibited $R_0$ values of less than 1, according to estimates in this study. This indicates that they had effectively curbed the transmission of COVID-19 during the period under study. Additionally, our research delved into the dynamic aspect of the pandemic by exploring the time-varying reproduction number. Among the top six countries with the highest confirmed COVID-19 cases, results demonstrated that community infections continued to drive the spread of the virus, even in the face of government intervention measures. This underscored the persistence of the challenge posed by community transmission.

The implications of these findings are profound and carry significant policy and public health relevance. Governments and policymakers can draw from this research to develop and implement effective control and preventive measures tailored to the unique dynamics of their respective regions for anticipated, similar future pandemics. In particular, in the future, countries with higher $R_0$ values should consider intensifying control and prevention efforts rather than prematurely relaxing restrictions. This could involve the expansion of widespread testing, rigorous contact tracing, and isolation/quarantine measures to curtail transmission. Furthermore, in line with guidance from the World Health Organization (WHO), these measures should be complemented by individual actions. These include adhering to social distancing protocols, avoiding large gatherings, practicing frequent hand hygiene through regular washing or sanitizing, staying at home whenever possible, and wearing masks in public places. Such collective and individual efforts are paramount to slowing down the outbreak and ultimately mitigating the impact of pandemics in sub-Saharan Africa.

## Study limitations

This study is not without limitations. First, we utilized data from the COVID-19 Data Repository by the Center for Systems Science and Engineering (CSSE) at Johns Hopkins University, a widely recognized and comprehensive source for global COVID-19 data. However, as with any secondary data source, there are inherent limitations and biases in the use of single data source. Variations in reporting practices, data collection strategies/methodologies, and the dynamic nature of the pandemic may have introduced potential biases. Hence, future research endeavors could benefit from incorporating additional data sources, exploring sensitivity analyses such as meta-analysis, to further validate and strengthen the robustness of our findings.

Secondly, in employing log-linear models and moment-generating functions to analyze our data, it is important to acknowledge potential biases and uncertainties that may influence the robustness of our statistical methods. One critical aspect deserving attention is the assumption underlying the serial interval distribution, which plays a pivotal role in our modeling approach. While we strove to choose a distribution that aligns with the characteristics of the disease under study, uncertainties persist in accurately capturing the true dynamics of transmission. Factors such as variations in reporting practices, the presence of asymptomatic cases, and the evolving nature of the virus itself may introduce biases in our estimations. Moreover, the inherent variability in individual behaviors and interventions across different populations and settings adds another layer of complexity. The assumed shape of the serial interval distribution may not fully capture the intricacies of transmission dynamics in diverse contexts, potentially leading to biased parameter estimates. To mitigate these concerns, future studies can explore alternative serial interval distributions and assess the robustness of our results.

Additionally, our analyses rely on the availability and accuracy of reported data, and any discrepancies or potential under-reporting could introduce bias into our estimates. Broadly speaking, under-reporting of cases is a pervasive challenge in the context of infectious disease surveillance. Variability in testing rates among countries, coupled with differences in reporting practices, may lead to discrepancies in the observed number of cases. The extent of underreporting may vary across regions, and the true magnitude of the pandemic could be underestimated in areas with limited testing capacity or reporting infrastructure. Hence, caution should be exercised in interpreting our results, and the estimates presented may not fully capture the actual scale of transmission. Moreover, the variability in testing rates among countries introduces a layer of complexity to our analyses. Disparities in testing accessibility and strategies can influence the observed trends and, consequently, the accuracy of the reproduction number estimates. It is thus crucial to recognize that our study relies on reported data, and the true dynamics of the pandemic may be obscured by these testing-related variations.

## Data Availability

The data supporting the findings of this study are sourced from the COVID-19 Data Repository by the Center for Systems Science and Engineering (CSSE) at Johns Hopkins University and is openly available at https://github.com/CSSEGISandData/COVID-19.

## Code availability

The code used for producing the figures in this article can be found in the GitHub Repository: https://github.com/IddiSam/Covid19SSA.git.

## References

[1] Anjorin AA (2020). The coronavirus disease 2019 (COVID-19) pandemic: a review and an update on cases in Africa. Asian Pac J Trop Med..

[2] Organization WH. COVID-19 Public Health Emergency of International Concern (PHEIC) Global research and innovation forum. 2023. https://www.who.int/publications/m/item/covid-19-public-health-emergency-of-international-concern-(pheic)-global-research-and-innovation-forum. Accessed 15 Mar 2020.

[3] Organization WH. WHO Coronavirus (COVID-19) Dashboard. 2023. https://covid19.who.int/?mapFilter=cases. Accessed 4 Oct 2023.

[4] Mehtar S, Preiser W, Lakhe NA, Bousso A, TamFum JJM, Kallay O (2020). Limiting the spread of COVID-19 in Africa: one size mitigation strategies do not fit all countries. Lancet Glob Health..

[5] Nkengasong JN, Mankoula W (2020). Looming threat of COVID-19 infection in Africa: act collectively, and fast. Lancet..

[6] Ma J (2020). Estimating epidemic exponential growth rate and basic reproduction number. Infect Dis Model..

[7] Zhang S, Diao M, Yu W, Pei L, Lin Z, Chen D (2020). Estimation of the reproductive number of novel coronavirus (COVID-19) and the probable outbreak size on the Diamond Princess cruise ship: A data-driven analysis. Int J Infect Dis..

[8] Van den Driessche P (2017). Reproduction numbers of infectious disease models. Infect Dis Model..

[9] Linka K, Peirlinck M, Kuhl E (2020). The reproduction number of COVID-19 and its correlation with public health interventions. Comput Mech..

[10] Chowdhury R, Heng K, Shawon MSR, Goh G, Okonofua D, Ochoa-Rosales C (2020). Dynamic interventions to control COVID-19 pandemic: a multivariate prediction modelling study comparing 16 worldwide countries. Eur J Epidemiol..

[11] Hens N, Shkedy Z, Aerts M, Faes C, Van Damme P, Beutels P. Modeling infectious disease parameters based on serological and social contact data: a modern statistical perspective, vol. 63. Springer Science & Business Media; 2012.

[12] Alimohamadi Y, Taghdir M, Sepandi M (2020). Estimate of the basic reproduction number for COVID-19: a systematic review and meta-analysis. J Prev Med Public Health..

[13] Liu Y, Gayle AA, Wilder-Smith A, Rocklöv J. The reproductive number of COVID-19 is higher compared to SARS coronavirus. J Travel Med. 2020. 10.1093/jtm/taaa021 PMC7074654 32052846

[14] Zhao S, Musa SS, Lin Q, Ran J, Yang G, Wang W (2020). Estimating the unreported number of novel coronavirus (2019-nCoV) cases in China in the first half of January 2020: a data-driven modelling analysis of the early outbreak. J Clin Med..

[15] Shim E, Tariq A, Choi W, Lee Y, Chowell G (2020). Transmission potential and severity of COVID-19 in South Korea. Int J Infect Dis..

[16] Dong E, Du H, Gardner L (2020). An interactive web-based dashboard to track COVID-19 in real time. Lancet Infect Dis..

[17] Nishiura H (2010). Correcting the actual reproduction number: a simple method to estimate R 0 from early epidemic growth data. Int J Environ Res Public Health..

[18] Wallinga J, Lipsitch M (2007). How generation intervals shape the relationship between growth rates and reproductive numbers. Proc R Soc B Biol Sci..

[19] Forsberg White L, Pagano M (2008). A likelihood-based method for real-time estimation of the serial interval and reproductive number of an epidemic. Stat Med..

[20] Cori A, Ferguson NM, Fraser C, Cauchemez S (2013). A new framework and software to estimate time-varying reproduction numbers during epidemics. Am J Epidemiol..

[21] Fraser C, Cummings DA, Klinkenberg D, Burke DS, Ferguson NM (2011). Influenza transmission in households during the 1918 pandemic. Am J Epidemiol..

[22] Li Q, Guan X, Wu P, Wang X, Zhou L, Tong Y (2020). Early transmission dynamics in Wuhan, China, of novel coronavirus-infected pneumonia. N Engl J Med..

[23] Han Q, Bragazzi N, Asgary A, Orbinski J, Wu J, Kong JD (2023). Estimation of epidemiological parameters and ascertainment rate from early transmission of COVID-19 across Africa. R Soc Open Sci..

[24] Oshinubi K, Rachdi M, Demongeot J. Analysis of reproduction number R0 of COVID-19 using current health expenditure as gross domestic product percentage (CHE/GDP) across countries. In: Healthcare, vol. 9. MDPI; 2021. p. 1247. 10.3390/healthcare9101247 PMC8535930 34682927

[25] Demongeot J, Oshinubi K, Rachdi M, Seligmann H, Thuderoz F, Waku J (2021). Estimation of daily reproduction numbers during the COVID-19 outbreak. Computation..

[26] Ofori SK, Schwind JS, Sullivan KL, Cowling BJ, Chowell G, Fung ICH (2022). Transmission dynamics of COVID-19 in Ghana and the impact of public health interventions. Am J Trop Med Hyg..

[27] Youdom SW, Tonnang HE, Choukem SP. Modelling and projections of the COVID-19 epidemic and the potential impact of social distancing in Cameroon. J Public Health Afr. 2021;12(2). 10.4081/jphia.2021.1479 PMC8819455 35136536

[28] Adekunle AI, Adegboye O, Gayawan E, McBryde E (2020). Is Nigeria really on top of COVID-19? Message from effective reproduction number. Epidemiol Infect..

[29] Jacobs ED, Okeke MI. A critical evaluation of Nigeria’s response to the first wave of COVID-19. Bull National Res Cent. 2022;46(1):44. 10.1186/s42269-022-00729-9 PMC8867460 35228791

